# Optimizing Primary Care Tools for Incontinence MAnagement (OPTIMA): protocol for a cluster randomized controlled trial

**DOI:** 10.1186/s13063-026-09427-7

**Published:** 2026-03-25

**Authors:** Maxwell B. Moore, Kyle Okamuro, Catherine Bresee, Ramy Eskander, Geneen T. Gin, Tamara Grisales, Anthony Galvez, Kimberly Gregory, Jejo Koola, Emily S. Lukacz, Allison M. Mays, Teryl K. Nuckols, Chaztyn Pangelina, Joshua Pevnick, David B. Reuben, Jennifer Singer, Ming Tai-Seale, Annie Wang, Neil Wenger, Shirley Wu, Tajnoos Yazdany, Xi Zhu, Jennifer T. Anger, the INTUIT-PC Research Group, Sharon Alonso, Rosa-Eugenia Carias-Veloz, Guillermo Castro, Sarah Conner, Stanislav Cuseac, Isabella Dolendo, Karyn Eilber, Elizabeth Hernandez, Janelly Jimenez, Carmen Mendez, Marlene Millen, Samina Saeb, Melissa Suarez, Florin Vaida, Katherine Ward, Kelly Williams

**Affiliations:** 1 Department of Urology, UC San Diego Health, San Diego, CA USA; 2 Biostatistics Shared Resources, Cedars-Sinai Medical Center, Los Angeles, CA USA; 3 Department of Obstetrics and Gynecology, UCLA Health, Los Angeles, CA USA; 4 Department of Obstetrics and Gynecology, Harbor-UCLA Medical Center, Los Angeles, CA USA; 5 Department of Family Medicine and Public Health, UC San Diego Health, San Diego, CA USA; 6 Department of Obstetrics & Gynecology, Cedars-Sinai Medical Center, Los Angeles, CA USA; 7 Department of Medicine, UC San Diego, San Diego, CA USA; 8 Department of Obstetrics, Gynecology, and Reproductive Sciences, UC San Diego School of Medicine, San Diego, CA USA; 9 Department of Medicine, Cedars-Sinai Medical Center, Los Angeles, CA USA; 10 Department of Biomedical Sciences, Cedars-Sinai Medical Center, Los Angeles, CA USA; 11 Multicampus Program in Geriatrics Medicine & Gerontology, UCLA Health, Los Angeles, CA USA; 12 Department of Urology, UCLA Health, Los Angeles, CA USA; 13 Department of Family Medicine, UC San Diego Health School of Medicine, San Diego, CA USA; 14 Iris Cantor UCLA Women’s Health Center, UCLA Health, Los Angeles, CA USA; 15 Department of Medicine, UCLA Health, Los Angeles, CA USA; 16 Department of Family Medicine, Harbor-UCLA Medical Center, Los Angeles, CA USA; 17 Department of Health Policy and Management, UCLA Fielding School of Public Health, Los Angeles, CA USA

**Keywords:** Urinary incontinence, Primary care, Practice-based intervention, Clinical decision support, Quality of care, Patient-centered outcomes

## Abstract

**Background:**

The burden of urinary incontinence (UI) on the American public is great. The impact of this condition will only continue to rise as the population ages, yet the quality of care provided at the primary care level has been inadequate to date. The Optimizing Primary Care Tools for Incontinence MAnagement (OPTIMA) study has been designed as a four-pronged, practice-based incontinence intervention aimed at improving the management of UI by primary care providers (PCP).

**Methods:**

In this pragmatic cluster randomized controlled trial, providers across four Southern California healthcare systems are randomized at the office level to receive incontinence intervention (*n* = 24 offices; 72 providers) vs. a non-intervention routine primary care cohort (*n* = 24 offices; 72 providers). The intervention includes (i) academic detailing with physician education and individual performance feedback; (ii) clinical decision support with note templates, order sets, and pop-up EHR alerts; (iii) access to co-management with a dedicated advanced practice provider; and (iv) implementation of an electronic referral service in which a specialist screens referrals for appropriateness. To achieve adequate power, the study will require 720 patients (360 patients per arm, average of 15 patients per office).

The primary provider outcome is the quality of UI care, as measured by adherence to a set of 13 quality indicators (QIs) on a 6-month chart review. Secondary provider outcomes include specialty referral rates (overall specialist referrals and presence of appropriate delay of referral) and disease-specific knowledge measured by the Pelvic Floor Awareness and Knowledge Survey (PFAKS). Patient self-reported outcomes include disease-specific knowledge measured by PFAKS, UI severity measured by the International Consultation on Incontinence Questionnaire (ICIQ-SF) and Urinary Distress Inventory (UDI-6), UI response to therapy measured by Patient Global Impression of Improvement (PGI-I), and patient-perceived shared decision making via the 9-item Shared Decision Making Questionnaire (SDM-Q-9). Patient outcomes are measured at baseline, 3-, and 6-month timepoints post-appointment with the study provider.

**Discussion:**

We expect this study to determine the efficacy and impact of comprehensive practice-based interventions on provider quality for UI care.

**Trial registration:**

The study is registered at ClinicalTrials.gov (NCT05534412). Registered on August 1, 2022.

## Administrative information

Note: the numbers in curly brackets in this protocol refer to SPIRIT checklist item numbers. The order of the items has been modified to group similar items (see http://www.equator-network.org/reporting-guidelines/spirit-2013-statement-defining-standard-protocol-items-for-clinical-trials/).

**Table:** 

Title {1}	*O*ptimizing *P*rimary Care *T*ools for *I*ncontinence *MA*nagement (OPTIMA): protocol for a cluster randomized controlled trial
Trial registration {2a and 2b}.	The study is registered at clinicaltrials.gov (NCT05534412). Registered on August 1, 2022.
Protocol version {3}	Feb 20, 2025, v77
Funding {4}	This trial is funded by the Agency for Healthcare Research and Quality (AHRQ)
Author details {5a}	Maxwell B. Moore, Department of Urology, UC San Diego Health, San Diego, CA, USA Kyle Okamuro, Department of Urology, UC San Diego Health, San Diego, CA, USA Catherine Bresee, Biostatistics Shared Resources, Cedars-Sinai Medical Center, Los Angeles, CA, USA Ramy Eskander, Department of Obstetrics and Gynecology, Harbor-UCLA Medical Center, Los Angeles, CA, USA Geneen T. Gin, Department of Family Medicine and Public Health, UC San Diego Health, San Diego, CA, USA Tamara Grisales, Department of Obstetrics and Gynecology, UCLA Health, Los Angeles, CA, USA Anthony Galvez, Department of Urology, UC San Diego Health, San Diego, CA, USA Kimberly Gregory, Department of Obstetrics & Gynecology, Cedars-Sinai Medical Center, Los Angeles, CA, USA Jejo Koola, Department of Medicine, UC San Diego, San Diego, CA, USA Emily S. Lukacz, Department of Obstetrics, Gynecology, and Reproductive Sciences, UC San Diego School of Medicine, San Diego, CA, USA Allison M. Mays, Department of Medicine, Cedars-Sinai Medical Center, Los Angeles, CA, USA Teryl K. Nuckols, Department of Medicine, Cedars-Sinai Medical Center, Los Angeles, CA, USA Chaztyn Pangelina, Department of Urology, UC San Diego Health, San Diego, CA, USA Joshua Pevnick, Department of Biomedical Sciences, Cedars-Sinai Medical Center, Los Angeles, CA, USA David B. Reuben, Multicampus Program in Geriatrics Medicine & Gerontology, UCLA Health, Los Angeles, CA, USA Jennifer Singer, Department of Urology, UCLA Health, Los Angeles, CA, USA Ming Tai-Seale, Department of Family Medicine, UC San Diego Health School of Medicine, San Diego, CA, USA Annie Wang, Iris Cantor UCLA Women’s Health Center, UCLA Health, Los Angeles, CA, USA Neil Wenger, Department of Medicine, UCLA Health, Los Angeles, CA, USA Shirley Wu, Department of Family Medicine, Harbor-UCLA Medical Center, Los Angeles, CA, USA Tajnoos Yazdany, Department of Obstetrics and Gynecology, Harbor-UCLA Medical Center, Los Angeles, CA, USA Xi Zhu, Department of Health Policy and Management, UCLA Fielding School of Public Health, Los Angeles, CA, USA Jennifer T. Anger, Department of Urology, UC San Diego Health, San Diego, CA, USA
Name and contact information for the trial sponsor {5b}	Sponsor: University of California, San Diego (UCSD), San Diego, CA, USA Primary Investigator (contact): Dr. Jennifer T. Anger, MD, MPH Department of Urology, UC San Diego Health janger@health.ucsd.edu
Role of sponsor {5c}	The sponsor maintains authority over all aspects of the trial including design, management, interpretation of results, and publication.

## Introduction

### Background and rationale {6a}

Urinary incontinence (UI) is defined by the International Continence Society as the complaint of any involuntary leakage of urine [1]. According to the National Health and Nutrition Examination Survey, the prevalence of UI among adult women ranges from 38% to 53% [2, 3]. With the aging population, it is projected that the number of women with UI will increase by 55% between the years 2010 and 2050 [4]. UI negatively impacts health-related quality of life and is particularly associated with embarrassment, stigma, and social isolation [5].

The Assessing Care of Vulnerable Elders (ACOVE) project previously developed indicators of high-quality care for vulnerable community-dwelling elders with UI [6]. Gnanadesigan et al. used these quality indicators (QIs) to measure care among 372 patients identified to be at risk for functional decline [7]. They found that the quality of UI care provided to vulnerable older patients was inadequate. Pelvic examination, an important measure, was performed in only 20% of older women with UI; only 50% of eligible patients received medical treatment for incontinence, and only 13% were prescribed behavioral intervention despite its proven effectiveness [7].

Poor quality of UI care is not unique to older adults. We compared care for the ACOVE population to that provided to 137 younger women with a diagnosis of UI in a hospital-based multi-specialty medical group in Los Angeles. UI care was insufficient in both groups [8]. Younger women, in particular, are more likely to be referred directly to an incontinence specialist — such as a urologist or urogynecologist — without receiving any incontinence care from their primary care provider (PCP) [2]. These factors lead to surgical specialists performing first-line care for UI, which is costly to the healthcare system and reduces access to specialty care for those who need higher-level UI care [9].

### Objectives and trial design {7} {8}

**Fig. 1:**
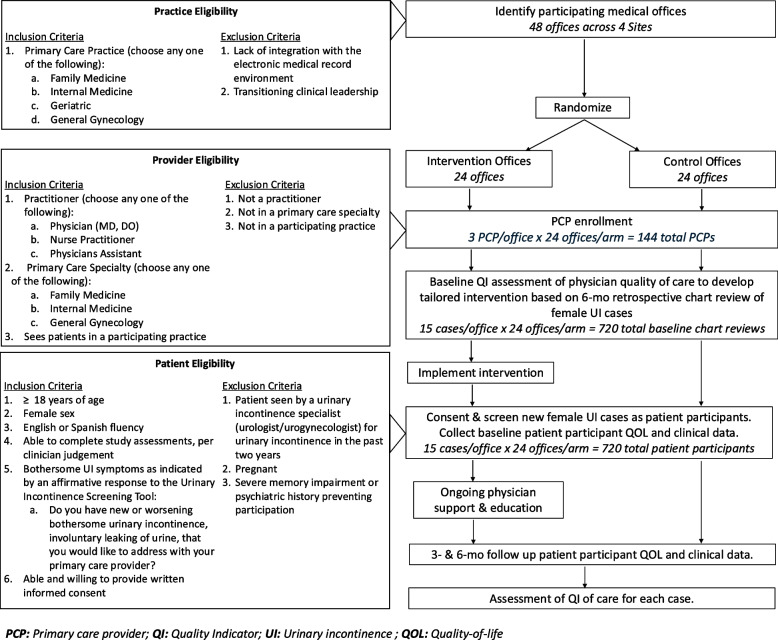
Study schematic and eligibility for practice, provider, and patient participants. Legend: Flowchart to the study with inclusion/exclusion criteria for each level of recruitment, including practice, primary care provider (PCP), and female patient with bothersome urinary incontinence (UI)

The Optimizing Primary Care Tools for Incontinence MAnagement (OPTIMA) project is a cluster randomized trial of a four-pronged practice-based incontinence intervention conducted across four health systems in Southern California (Fig. 1). It was created in response to the Agency for Healthcare Research and Quality (AHRQ) initiative: Improving Nonsurgical Treatment of Urinary Incontinence among Women in Primary Care (RFA-HS-21-001). The overall goals of the project are to improve primary care management of incontinence, reduce unnecessary referrals to specialists, and improve patient-centered outcomes. Herein, we describe the detailed methods of this clinical trial, which is reported in accordance with the SPIRIT 2013 (Standard Protocol Items: Recommendation for International Trials) guidelines [10].

## Methods

### Study setting {9}

The present study is a cluster randomized design with a target of 48 offices in Southern California from 4 academic health systems: one private, two public-private, and one public county health system.

### Eligibility criteria {10}

Inclusion and exclusion criteria for each level of recruitment (practice, PCP, and patient) are described in Fig. 1.

### Informed consent {26a} {26b}

Informed consent for PCP cohort is obtained by a member of the study team at respective sites. After single IRB approval was obtained (IRB #802004), the study team obtained a waiver of documented consent and a full HIPAA waiver at each site, allowing patients to self-enroll in the study. In addition, the study team obtained a waiver of informed consent and a partial HIPAA waiver for baseline chart abstraction of prior UI cases.

## Interventions

### Explanation for the choice of comparators {6b}

Eligible practices (i.e., offices) identified by health system and primary care leadership were randomized into intervention and routine care arms (Fig. 1). Office specialties include family medicine, gynecology, geriatrics, and general internal medicine, as they manage women’s primary care and often show a need in the literature for improved UI care.

The comparator is usual (standard-of-care) UI management as delivered at each participating site. Usual care refers to the care that individual clinicians provide based on their individual training. The usual care arm PCPs receive a best practice alert when they open the patient encounter, but no additional UI-specific tools, training, or decision support are provided.

### Intervention description {11a}

**Fig. 2:**
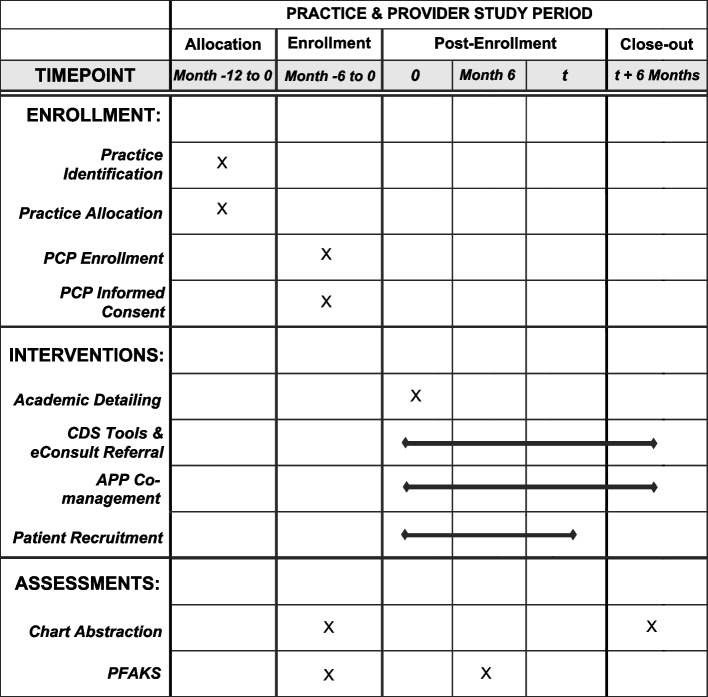
SPIRIT diagram of practice and PCP timeline. Legend: Allocation occurs at practice-level prior to PCP enrollment. Timepoint (*t*) at which PCP’s final patient participant is enrolled differs based on individual provider

The four-pronged, practice-based incontinence intervention includes (1) provider education and training, (2) electronic clinical decision support tools, (3) opportunity for co-management with an advanced practice provider (APP), and (4) access to an electronic referral mechanism in which a specialist screens referrals for appropriateness. Outcomes to be measured include quality of provider care (defined by adherence to urinary incontinence QIs as determined by 6-month chart review) (Table 1), specialist referral rates, and patient-reported outcomes. Practice and PCP timeline detailed in Fig. 2.

**Table 1:** Urinary incontinence quality indicators (QIs)

Evidence-based measures of implementation strategies to be used as care guidelines and care measurement tools	Evidence base
*Diagnosis: targeted evaluation/basic history*	
1) A basic history should be obtained from a woman presenting with complaints of new or worsening bothersome UI, including: a) Determining whether stress, urge, or both symptoms are present b) Lifestyle factors (fluid intake) if urge urinary incontinence (UUI) is present 2) History of prior pharmacologic therapy for UI 3) Severity assessment	AUA/SUFU Guideline [11] AHRQ Update [12] Delphi Panel [13]
*Targeted physical exam*	
4) A physical exam should be performed on a woman presenting with complaints of new/worsening bothersome UI symptoms, including a vaginal exam to assess for contributors to UI (fibroids, pelvic organ prolapse) and an assessment of pelvic floor muscle strength (ability to perform Kegel exercises)	LA County DHS SUI Expected Practices Delphi Panel [13]
*Diagnostic testing*	
5) Urinalysis should be performed on a woman who presented with new/worsening bothersome SUI to screen for microhematuria or urinary tract infection	LA County DHS SUI Expected Practices Delphi Panel [13]
*Treatment/management UUI*	
Behavioral therapy 6) A woman who presents with new or worsening bothersome UI should initially be offered pelvic floor muscle training (PFMT)	ACP clinical practice guidelines [14] AUA/SUFU Guideline [11] AHRQ Update [12] Delphi Panel [13] Cochrane review [15]
*Treatment/management of SUI*	
Behavioral therapy 7) A woman who is overweight (BMI > 25) with new or worsening symptoms should be advised to lose weight Pharmacological therapy 8) Anticholinergic or beta agonist therapy should not be offered as a treatment to a woman who presents with new or worsening bothersome SUI without symptoms of OAB	ACP clinical practice guidelines [14] Delphi Panel [13]
*Treatment/management of UUI*	
Pharmacological therapy 9) Anticholinergic or beta agonist therapy may be offered as a treatment to a woman who presents with new or worsening OAB Behavioral therapy 10) If a woman presents with new or worsening bothersome UUI, then a history about fluid intake should be obtained	AUA/SUFU Guideline [11]LA County DHS Expected Practices Delphi Panel [13]
QIs for urinary incontinence (UI) based on those developed by the Assessing Care of Vulnerable Elders (ACOVE) project	

*UUI *urge urinary incontinence, *SUI* stress urinary incontinence, *OAB* overactive bladder

#### Academic detailing

Enrolled PCPs receive academic detailing prior to patient recruitment. This portion of the intervention integrates principles of Educational Outreach, utilizing stepwise application of evidence-based techniques (i.e., baseline knowledge inventories, targeted interventions, etc.) to improve clinical decision-making [16]. First, PCPs complete their baseline UI knowledge assessment via the Pelvic Floor Awareness and Knowledge Survey (PFAKS), a 31-item validated pelvic floor disorder knowledge questionnaire [17]. Once complete, the study team holds an in-person or virtual lecture at intervention practices. Providers unable to attend receive a one-on-one lecture via Zoom. Lectures are led by a UI clinical champion, either a urologist/urogynecologist or PCP from that site who is part of the research team and has expertise in UI management. The lecture centers on a set of thirteen previously developed and tested quality-of-care indicators (QIs) that cover all aspects of non-surgical management of UI, including history and physical examinations (i.e., pelvic exam), urinalysis, initiation of conservative treatment (i.e., lifestyle/behavioral interventions, pelvic floor training, medication, and when to refer to a UI specialist in urogynecology (Table 1)). The QIs were independently developed using the RAND Appropriateness, or Delphi, Method [13] and are validated for use in a diverse population [8, 18, 19]. PCPs also receive a pocket guide with a treatment algorithm.

Within a month after the lecture, UI specialists provide individualized coaching for intervention PCPs. This includes a one-on-one review of the PCP’s baseline quality of care for UI, as measured by retrospective chart abstraction of up to 5 previous UI encounters. Baseline charts are identified by a diagnosis code for UI (Appendix A). After reviewing prior care, the UI specialist offers individualized recommendations. Intervention PCPs are then ready for patient recruitment. Baseline chart abstraction is also performed in the routine care cohort, although without the individualized coaching sessions.

#### Clinical decision support tools

Clinical decision support (CDS) refers to a range of tools and systems that help healthcare providers make informed decisions about patient care. The study team developed a combination of CDS tools that are implemented into each intervention office’s electronic health record (EHR) system. These included note templates (pre-formatted EHR clinical notes containing text and placeholders that streamline documentation and prompt providers to record and address key clinical information), order sets (pre-configured groups of laboratory tests, medications, and referrals that can be selected together to streamline ordering), and electronic alerts (automated, EHR-based prompts that notify providers when specific clinical criteria are met and suggest appropriate next steps) (Table 2). PCPs are taught how to utilize the CDS tools during the UI lecture.

**Table 2:** Clinical decision support tools

Quality indicators for which clinical decision support will be used	Type of clinical decision support
Diagnosis: targeted evaluation/basic history	
Positive screen	*Banner/smart zone*
1–3. Assessment of UI (symptoms and history)	*Templated note*
4. Targeted physical exam	*Templated note*
5. Diagnostic testing (Urinalysis)	*Order set*
6. Pelvic floor muscle exercises	*Templated note and order set for PFMT*
Treatment/management of SUI	
7. Weight loss recommended	*Templated note*
8. Anticholinergic (or beta agonist) therapy should not be offered as a treatment to a woman who presents with new or worsening bothersome SUI without symptoms of overactive bladder (OAB)	*Interruptive alert*
Treatment/management of UUI	
9. History about fluid intake should be obtained	*Templated note*
10. Counseling about behavioral modification, including fluid restriction and bladder training	*Templated note*
11. A woman with UUI/OAB who is prescribed anticholinergic medications should also be counseled about behavioral therapy	*Templated note and order set*
12. Medication (anticholinergics or beta-3 agonists) should be offered when behavioral therapy and PFMT do not control symptoms	*Templated note and order set*
Follow-up after treatment	
13. A woman treated for any form of UI should be re-evaluated within 3 months of initiating intervention for the efficacy and/or complications of any intervention	*Templated note and order set*

Electronic alerts for providers vary by site and include either an interruptive alert or a non-interruptive alert (banner). Electronic alerts are timed to fire within the EHR system during the PCP’s visit with patient participants, alerting the PCP to the patient’s positive screen for bothersome UI. Electronic alerts are the only aspect of the CDS toolset provided to both arms of the study. Since the primary outcome of interest is the quality of UI care provided (and not whether or not a UI-related conversation occurs in the first place), electronic alerts are implemented since embarrassment may contribute to patients’ reluctance to initiate UI conversations.

#### eConsult referrals

The study team implemented an electronic referral system at intervention offices modeled after the participating public county system’s electronic consultation system (eConsult). A specialist in urology or urogynecology triages referrals for appropriateness of care; for referrals where adequate care is not performed (i.e., one or more of the basic QIs for first-line UI care are not met), the reviewing specialist sends a direct message to the PCP with clinical recommendations to consider before referring to the specialist.

#### APP co-management

Intervention PCPs also have the option to refer study patients to an Advanced Practice Provider (APP) co-manager with expertise in UI. If referred, the patient is scheduled for a visit (telemedicine or in-person) with the APP, who provides follow-up teaching and non-surgical management according to standard clinical practice. APPs can also refer participating patients to a urology/urogynecology specialist as clinically indicated.

### Criteria for discontinuing or modifying allocated interventions {11b}

Enrolled PCPs who leave participating practice or health system will be excluded from the study. Patients can request to discontinue participation in the study at any point by informing their PCP and/or a member of the study team.

### Strategies to improve adherence to interventions {11c}

PCPs received a small honorarium for participating. Those who attend the lecture as part of the intervention arm also receive Maintenance of Certification (MOC) credit. PCP clinical champions periodically email the intervention PCPs to inquire about any questions they may have and assess their experience with the intervention toolset. These touchpoints also enable PCPs to discuss barriers and potential issues related to the study or the CDS/referral tools that arise as they see patient participants.

### Relevant concomitant care permitted or prohibited during the trial {11d}

PCPs in both arms manage patient care without prohibitions, including the option of referring to a specialist.

### Provisions for post-trial care {30}

PCPs in routine care offices know they will receive the intervention once their patient participation is complete (delayed intervention). Once the last patient participant for a routine care PCP has reached the 6-month timepoint in the study, routine care providers will be offered access to the intervention tools.

### Outcomes {12}

#### Primary outcome: quality-of-care measures

The primary outcome of this trial is provider quality of care. Provider quality of care in both arms is measured via retrospective chart abstraction at baseline and again for each respective patient participant. Care provided over a 6-month timeframe is evaluated for adherence with 13 primary care UI QIs we previously developed, to allow adequate time to comply with the QIs [8] (Table 1). For each chart abstraction, a given eligible indicator is scored as a dichotomous measure (met vs. not met). Aggregate scores are measured as proportions: encounters where patients receive the recommended care vs. total encounters where patients are eligible for specific QIs. The results will be reported as percentages ranging from 0 to 100%.

#### Secondary provider outcomes

Data on referral rates are also collected at the 6-month chart abstraction to assess overall specialty referral rates and the presence of an appropriate delay of referrals, in which referrals are placed only after first-line treatment is initiated.

**Fig. 3:**
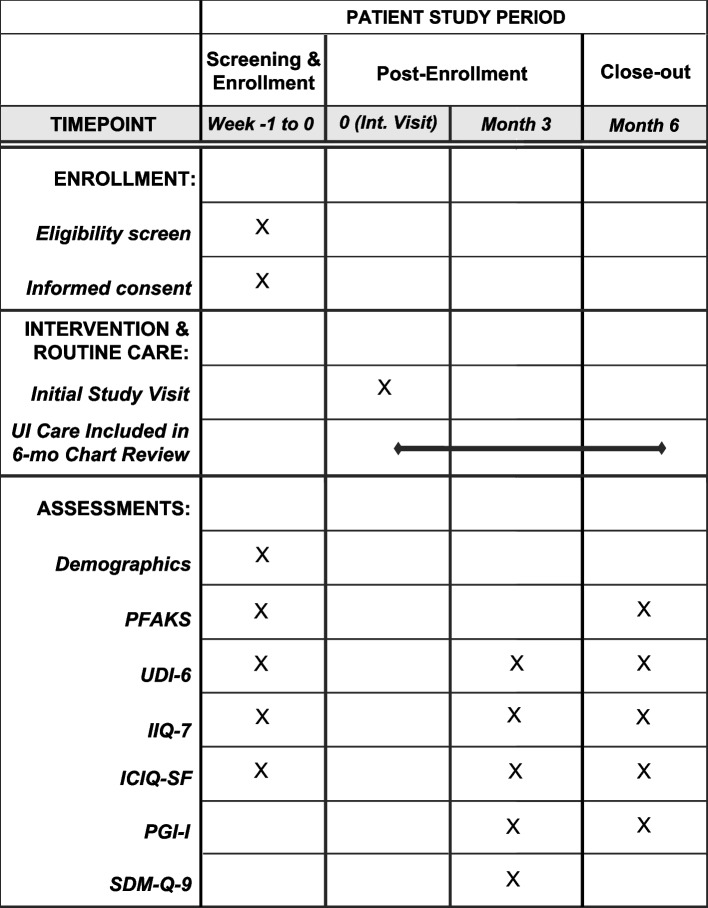
SPIRIT diagram of patient participant timeline (same for both arms). Legend: Allocation omitted, as randomization occurs at office level and patients are recruited from PCP upcoming visit

At baseline, providers complete a demographic questionnaire and a modified version of the Pelvic Floor Awareness and Knowledge Survey (Fig. 3). The PFAKS is a 31-item validated questionnaire assessing knowledge of three pelvic floor disorders: pelvic organ prolapse (11 items), stress urinary incontinence (SUI) (10 items), and overactive bladder (OAB) (10 items) with questions on condition pathophysiology, management, and quality of life for these conditions [17]. The modified PFAKS excludes the 11 pelvic organ prolapse items.

#### Patient reported outcomes

At baseline, all patient participants complete demographic questionnaires (Fig. 3). Symptom severity and disease-specific quality of life (QoL) are assessed with the Urinary Distress Inventory (UDI-6), Incontinence Impact Questionnaire, Short Form (IIQ-7), and the International Consultation on Incontinence Questionnaire-Urinary Incontinence Short Form (ICIQ-SF) [20, 21]. The UDI-6 (6-item, 5-point Likert scale) and IIQ-7 (7-item, 4-point Likert scale) are validated questionnaires measuring UI impact on QoL with a range of 0 to 100. The ICIQ-SF (6-item, 5-point Likert scale) is a validated questionnaire assessing symptom severity and QoL with a range of 0 to 21.

Patients also complete the following: the Patient Global Impression of Improvement (PGI-I), a single-item measure for symptom response to therapy [22]; the 9-Item Shared Decision Making Questionnaire (SDM-Q-9), assessing patient-perceived involvement in the clinical decision-making process via both Likert scale (range of 0 to 100) and free-response sections [23]; and lastly, the same modified PFAKS given to providers. A summary of the patient assessment timeline is shown in Fig. 3.

Spanish translations for all surveys are available for Spanish-speaking patients. The ICIQ-SF, UDI-6, and SDM-Q-9 have been validated in Spanish [24–26]. The PGI-I and modified PFAKS questionnaires have been translated into Spanish by certified translators, and the Spanish translation of the PFAKS is currently undergoing validation.

### Participant timeline {13}

Intervention PCPs receive the academic detailing portion of the intervention prior to patient recruitment. Six-month PFAKS is timed based on their office’s UI lecture (Fig. 3). Patient recruitment opens for intervention PCPs once their individual coaching session (i.e., lecture followed by a review of prior care with a clinical champion) is completed and is closed once 10–13 patients per provider are enrolled; the exact timeline will differ between PCPs based on factors such as their patient population (e.g., proportion eligible) and number of outpatient visits per week. Since routine care PCPs do not receive academic detailing, 6-month PFAKS will be timed based on the enrollment date. Patient recruitment in this arm begins once enrolled PCPs complete the baseline surveys. Both patients and PCPs in the routine care arm complete an identical battery of surveys as those in the intervention arm.

Patient participants are enrolled 1–2 weeks prior to an upcoming appointment with their PCP and complete the baseline survey battery upon enrollment. Patient participation includes the completion of baseline, 3-, and 6-month surveys; a patient is considered finished with the study once they complete the 6-month surveys (Fig. 3).

### Sample size {14}

All power estimates were performed assuming mixed-model regression modeling to account for the nested study design (patients within providers within offices) using PASS 2020 software [27]. As randomization will occur at the office level, power was computed assuming an average of 3 providers per office with an intra-cluster correlation of 0.04 (consistent with previous reports for process-of-care outcomes [28]) and a correlation of 0.16 across patients within each provider. Preliminary data found the average baseline quality of care to be 40% with a standard deviation of 20% [8, 18]. Therefore, to detect a change of 6% in total scores at an alpha level of 0.05 with 80% power, the study will require at least 720 patients from 48 offices (360 patients from 24 offices per study arm; see Fig. 1).

We conservatively estimate this sample size can detect a minimum odds ratio of 0.52, which is equivalent to a decrease in referral rates from 60 to 44% between the two treatment arms of the study, with 80% power at the 0.05 significance level. For patient-reported outcomes, our preliminary data had an average score of (± SD) of 35 (± 20) for the UDI-6 and 18 (± 20) for the IIQ-7. The study is powered to identify a difference of 6 points or greater in each of the two measures between study arms.

### Recruitment {15}

Prior to PCP recruitment, the study team met with health system and primary care leadership at each participating site to identify and select eligible primary care offices, with permission from health system, primary care, and practice leadership. Eligible PCPs that wished to participate were then recruited by the study team during individual office meetings.

For patient recruitment, a data analyst generates a weekly list of adult women with upcoming appointments in the next 10–14 days. One week before the scheduled appointments, the research team emails eligible patients the screening survey. Patients who answer that they (1) have new or worsening bothersome UI that they would like to address with their PCP and (2) have not been seen by a urologist or urogynecologist for UI in the past 2 years are then presented with the consent form from the same screening survey.

Because this is a pragmatic trial, variation in screening is allowed based on the population served. For example, to improve recruitment from a safety-net hospital with a large Spanish-speaking patient population with varying internet access, participants from this health system are recruited via a telephone-based screening performed by a fluent Spanish-speaking research team member.

## Assignment of interventions: allocation

### Sequence generation {16a}

Offices were assigned to study arms using randomization lists generated using Wei’s Urn method (to allow for equal sample sizes). Randomization at the practice level was done for all four sites by the study biostatistician 1:1 to each study arm, stratified by study site and office specialty (family medicine, gynecology, geriatrics, and general internal medicine), using PASS 2020 software [27]. Physicians working within a given practice received the same designation to prevent cross-contamination of intervention materials.

### Concealment and implementation {16b} {16c} {17a} {17b}

Office-level allocation was recorded on site-specific Real Electronic Data Capture (REDCap) servers (a password-protected data management platform). Individual PCPs were not randomized independently. Rather, once an office was assigned to a study arm, all PCPs recruited from that office inherited the office-level allocation. Office-level allocation was concealed from PCPs during recruitment.

Recruitment of PCPs from allocated offices was conducted by site-specific clinical champions, with assistance from trained research coordinators using standardized recruitment scripts that did not reference intervention assignment, thereby preventing inadvertent disclosure. PCPs were informed prior to enrollment that randomization occurred at the office level; however, they were not informed of their specific office’s assignment until after enrollment was complete. Though not provided as part of the enrollment process, patients can be informed of their PCP’s allocation provided upon request post-enrollment.

## Data collection and management

### Data collection {18a}

Trained research staff conduct the chart abstractions considering all of the patient’s records when assessing whether the patient is eligible for and receives the indicated care [29]. Individual site PIs review QIs that require a detailed clinical assessment. Baseline and 6-month chart abstraction data are collected and stored via REDCap eCRF. Patient and PCP self-report data are collected via REDCap survey.

### Participant retention {18b}

Plans to promote patient participant retention include tiered gift card incentives, with increasing value over time, as well as multiple reminder contacts via email and telephone to encourage survey completion.

PCP retention is supported through engagement of site-specific clinical champions, provision of CME credit contingent on completion of the full study protocol, and periodic email check-ins.

For patient participants who discontinue or deviate from the intervention protocol, all data collected up to the point of discontinuation are retained. In addition, clinical outcome data continue to be collected via chart abstraction under existing informed consent and HIPAA authorization, even if survey participation ceases.

Loss to follow-up between baseline and 6-month assessments will be monitored, and dropout rates will be compared between study arms. Where follow-up data are missing, multiple imputation techniques will be considered as appropriate, and missing data may alternatively be recorded as a separate category for modeling.

### Data management, security, and quality {5d} {19}

All study data are entered and managed using REDCap, by trained study personnel using standardized electronic forms with predefined coding schemes. Built-in validation rules, including logic checks and range checks, are implemented to promote data completeness and accuracy at the point of entry.

Data quality is overseen by the DCC through routine review for completeness, internal consistency, and cross-site variability. For chart-based data abstraction, standardized training procedures and periodic cross-checks are used to promote inter-rater consistency, with discrepancies resolved through consensus review involving site investigators as needed.

### Confidentiality {27}

All patient and PCP data is housed on site-specific REDCap servers only accessible to respective site’s study team through role-based permissions. To protect confidentiality, patient and provider data are deidentified at the time of entry and labeled only with a REDCap-generated unique study identifier. No direct personal identifiers (e.g., name, medical record number, date of birth) are stored in the analytic dataset.

Linkage between the study identifier and identifiable information, when required for study operations (e.g., recruitment tracking or follow-up), is maintained locally at each site in a secure, access-restricted file and is not shared across sites. Any data shared between sites is deidentified.

### Plans for collection, laboratory evaluation and storage of biological specimens for genetic or molecular analysis in this trial/future use {33}

No biological specimens are collected/evaluated in this protocol.

## Statistical methods

### Statistical methods for primary and secondary outcomes {20a} {20b}

The primary outcome of quality of care will be measured by constructing aggregate QI scores ranging from 0 to 100%, as described by McGlynn et al. [30]. 5% of all records will be re-abstracted to evaluate inter-rater reliability using Gwet’s AC1 correlation [31, 32]. Given the potential differences in patient and provider demographics between the private, public-private, and public county health systems, factors such as patient race/ethnicity, provider sex, and provider specialty will be assessed via a simplified mixed model regression, incorporating random effects to account for clustering. Analysis will be conducted using SAS with significance set at *p* = 0.05.

A chi-square test will be used to compare patient enrollment between health systems to evaluate the potential impact and efficacy of phone vs. email screening as a recruitment tool. Patient referral rate to specialty care in both intervention and routine care arms will be assessed using cluster logistic regression. Appropriate delay of referral is measured as a dichotomous outcome (timely vs. premature referral) with proportion between groups analyzed via a chi-squared test.

### Interim analyses {21b}

Not applicable.

### Methods for additional analyses (e.g., subgroup analyses) {20b}

Between-group differences across sites will be assessed for primary and secondary outcomes via subgroup analyses. All analyses will be conducted using SAS, with statistical significance set at *p* = 0.05. Adjustments for multiple comparisons will be applied where necessary.

### Methods in analysis to handle protocol non-adherence and any statistical methods to handle missing data {20c}

PCPs are given a 6-month window for each patient participant to address their UI. If no UI-related discussion or treatment occurs within this timeframe, the patient’s UI is considered an “unaddressed case.” This can be factored into PCP’s aggregate score by adding to the total number of visits with the patient to the denominator for their proportion of encounters where QI met vs. encounters eligible for QI. Unaddressed cases are also recorded for potential inclusion as secondary outcomes or in subgroup analyses, depending on trial outcomes.

### Plans to give access to the full protocol, participant level-data and statistical code {31c}

The full protocol and participant-level data are available from the relevant researchers upon request after the publication of the study results.

## Oversight and monitoring

### Composition of the coordinating center and trial steering committee {5d}

Day-to-day study activities are carried out by site research coordinators overseen by respective site-specific PIs, who are responsible for local study conduct, regulatory compliance, and protocol adherence. Each participating site holds internal monthly study meetings involving site PIs, research coordinators, and key clinical staff.

Study sponsor site acts as coordinating center, providing overall scientific, operational, and regulatory oversight across all participating sites. The coordinating center is led by the overall principal investigator and includes co-investigators (including informaticist), project manager, and research coordinators. This group functions as the trial steering committee and meets monthly via all-site teleconferences that include representation from each participating site.

Data coordination is overseen by the DCC, housed at the sponsor site. Patient- and provider-level data are collected and stored on site-specific REDCap servers accessible only to the local study teams. De-identified data are transferred to the coordinating center on a quarterly basis. The coordinating center reviews these data for quality.

An external advisory board provides independent oversight and meets semi-annually to review study progress and advise on implementation challenges.

### Composition of the data monitoring committee, its role and reporting structure {21a}

Not applicable due to the low-risk nature of the study.

### Adverse event reporting and harms {21a} {22}

Reliance on sponsor site IRB (IRB #802004) was approved at all four study sites. All adverse events reported to the study team will be recorded. Adverse events may be identified through patient self-report, provider report, or review of clinical and study data. Each participating site follows its local IRB policies for adverse event documentation and initial reporting. Under the single-IRB reliance agreement, all reportable adverse events are submitted to the reviewing IRB. Adverse events and unanticipated problems are reviewed by the study investigators, and relevant findings are communicated through established study oversight mechanisms, including regular investigator meetings and the External Advisory Board. Any events that are immediately addressed are by research staff.

### Frequency and plans for auditing trial conduct {23}

Adherence to regulatory compliance is carried out annually by each site IRB.

### Plans for communicating important protocol amendments to relevant parties (e.g. trial participants, ethical committees) {25}

Amendments to the trial protocol are submitted to the single IRB by the sponsor site PI. Approved protocols with tracked changes are submitted to the research team at each study site for submission of amendments.

### Dissemination plans {31a}

Study results will be published in a peer-reviewed journal upon completion of the trial and will be reported in concordance with the CONsolidated Standards of Reporting Trials (CONSORT) 2010 Guideline [33, 34]. Results will also be disseminated via presentation at scientific meetings. Once initial study results are published, members of the INTUIT-PC Research group can disseminate further analyses through peer-reviewed publication and scientific meetings. Dissemination efforts with the National Association For Continence (NAFC) will aim to utilize preexisting digital and organizational information-sharing infrastructure to disseminate research findings with patients and providers nationally.

## Discussion

The methods in this trial are novel for several reasons. First, we will bring subspecialty expertise to primary care settings, improving on interventions that have been done previously. Second, we have established a pragmatic approach for incorporating subspecialty expertise in primary care settings that does not overburden primary care. Third, we seek to imbed components of the intervention into the electronic medical record, a needed element which will likely have a much greater impact on quality than prior interventions [35–37]. Fourth, we will learn from the successful electronic consultation system already in place in the Los Angeles County healthcare system as we implement an electronic consultation system in the three non-county sites [38]. And lastly, we will apply an Advanced Practice Provider (APP) co-management strategy in that APPs will conduct the patient education portion of the intervention for providers who wish to have that support. APP co-management has been shown to have a greater impact on quality than other educational measures, and reduces the work needed on the part of PCPs [39, 40]. APP co-management will also leverage the recent COVID-19 related implementation of telemedicine, adding to the feasibility of this approach.

The primary strength of the OPTIMA study is its design as a large, pragmatic, cluster randomized clinical trial using rigorous methodology across various Southern California health systems, including one private, two public-private, and one public county academic health system. One of the CDS tools reminds providers to discuss UI with their patients *during their visit* to assist providers in addressing UI if patients are reluctant to bring up their symptoms due to embarrassment or stigma. We expect this tool to significantly increase the likelihood of a conversation about UI occurring between patient and provider. In addition, implementation of EHR alerts across both arms ensures that all providers are informed of UI in a similar manner, thereby increasing the likelihood that any between-group differences are due to variation in the intervention, rather than screening practices. Furthermore, prior work by Assessing Care of Vulnerable Elders (ACOVE) suggests that screening patients and alerting providers of a positive screen will have little impact on the quality of incontinence care provided [7].

Hispanic women experience higher rates of stress and mixed UI compared to their non-Hispanic counterparts [41], yet often receive suboptimal care compared to other ethnic groups. Past analysis of Medicare data (1999–2001) also revealed disparities in that Caucasian and Hispanic women with a diagnosis of stress UI were more likely to undergo sling surgery than black or Asian women. Furthermore, Spanish-speaking patients tend to have less understanding of their condition and greater anxieties and resistance regarding treatment options offered compared to their English-speaking counterparts [42]. As such, the study team placed significant care during the design and implementation of the study to ensure substantial recruitment from this patient population. This includes the addition of phone-based screening with Spanish-speaking research team members and formulation and validation of Spanish versions for all questionnaires. However, despite these efforts, potential limitations remain when assessing such process-of-care outcomes due to structural and cultural factors outside the realm of individual providers.

As a multi-institution pragmatic study, there are potential limitations to the study’s execution, such as the lack of blinding of PCPs, patient participants, and study staff. However, we have reasons to believe this design feature will not significantly bias the results: office-level randomization limits avenues for cross-contamination of educational material and CDS tools, and providers in routine care offices have the opportunity to receive a delayed intervention. Although chart abstractors cannot be blinded given the makeup of the EHRs, their methods of QI assessment are strictly standardized, with detailed scoring rubrics and protocols developed to limit the potential bias and ensure inter-rater reliability.

Our assessment of incontinence outcomes is purposefully broad and encompassing, as we aim to measure and combine outcomes of different non-surgical treatments for stress and urge UI. We have excluded objective outcome measures, such as voiding diaries or pad tests, as we seek to minimize the burden to patients and providers. However, our study is nonetheless powered to detect a clinically meaningful difference in outcomes pertinent to patients between intervention and routine care groups, despite variation in treatment, case mix, and patient demographics.

Results of this study will contribute to the translation of patient-centered outcome research into clinical practice and improved screening and quality of care for women struggling with UI. After the intervention is complete, we will focus on dissemination of these tools and broad implementation of electronic clinical decision support tools across healthcare systems.

## Trial status

Patient recruitment is currently underway, and outcomes data collection for both patients and PCPs is estimated to be complete by the end of 2025.

## Data Availability

The datasets used and/or analyzed during the current trial are available from the corresponding author on reasonable request.

## Abbreviations

APP: Advanced practice provider
CDS: Clinical decision support
EHR: Electronic health record
ICIQ-SF: International Consultation on Incontinence Questionnaire, Short Form
IIQ-7: Incontinence Impact Questionnaire, Short Form
PCP: Primary care provider
PFAKS: Pelvic Floor and Knowledge Survey
PGI-I: Patient Global Impression of Improvement
QI: Quality indicator
SDM-Q-9: 9-Item Shared Decision Making Questionnaire
UDI-6: Urogenital Distress Inventory, Short Form
UI: Urinary incontinence
